# A simple and fast method to exclude high *Plasmodium falciparum *parasitaemia in travellers with imported malaria

**DOI:** 10.1186/1475-2875-10-300

**Published:** 2011-10-14

**Authors:** Tom van Gool, Marlies E van Wolfswinkel, Rob Koelewijn, Pieter PAM van Thiel, Jan Jacobs, Jaap J van Hellemond, Perry JJ van Genderen

**Affiliations:** 1 Department of Parasitology, Academic Medical Center, Amsterdam, The Netherlands; 2 Department of Internal Medicine, Harbour Hospital, Rotterdam, The Netherlands; 3 Department of Parasitology, Harbour Hospital, Rotterdam, The Netherlands; 4 Department of Internal Medicine, Academic Medical Center, Amsterdam, The Netherlands; 5 Department of Clinical Sciences, Institute of Tropical Medicine, Antwerp, Belgium; 6 Department of Medical Microbiology and Infectious Diseases, Erasmus University Hospital, Rotterdam, The Netherlands; 7 Institute for Tropical Diseases, Harbour Hospital, Rotterdam, The Netherlands

**Keywords:** malaria, traveller, imported disease, aldolase, severe malaria, rapid diagnostic test

## Abstract

**Background:**

Counts of malaria parasites in peripheral blood are important to assess severity of *Plasmodium falciparum *malaria. Thin and thick smears are routinely used for this purpose.

**Methods:**

In this study the Binax NOW^® ^Malaria Test, an easy-to-perform rapid diagnostic test, with Histidine Rich Protein-2 (HRP-2) and aldolase as diagnostic markers, was used for semi-quantitative assessment of parasitaemia of *P. faciparum*.

**Results:**

In 257 patients with imported *P. falciparum *malaria, reactivity of aldolase increased with higher parasitaemia. In all patients with a parasitaemia above 50,000 asexual parasites/μl (> 1%) co-reactivity of HRP-2 and aldolase was observed. Absence of aldolase reactivity in the presence of HRP-2 was a reliable predictive marker to exclude high (> 1%) parasitaemia in *P. falciparum *malaria.

**Conclusions:**

Assessment of HRP-2 and aldolase co-reactivity can be of help in clinical decision making in the acute care setting of returning travellers suspected of having malaria.

## Background

The global burden of malaria is largely carried by the world's malaria-endemic regions with as many as 225 million cases and a death toll of more than 750,000 individuals in 2009 [1]. In striking contrast, in non-endemic industrialized countries malaria is seen as an occasionally imported disease in non-immune travellers, but it still represents a potentially fatal disease [2,3]. Without prompt and proper treatment malaria may rapidly progress to complications and even death. Hence, all patients must be assessed for signs or symptoms suggestive of an increased risk for complications. Due to unfamiliarity with the disease in non-endemic countries, ill-returning travellers frequently present to physicians who have no tropical medicine expertise and to primary health care facilities that lack expert diagnostic capabilities. As a result, diagnosis of malaria may be delayed or even missed, resulting in more severe disease or even fatalities [4,5].

Recent studies in non-endemic industrialized countries showed that rapid diagnostic tests (RDTs) for malaria provide an excellent tool for diagnosis of malaria as compared to peripheral blood smears [6]. Although highly sensitive in diagnosing *Plasmodium falciparum *malaria, RDTs are not thought to provide sufficient information about parasitaemia, one of the major determinants of disease severity [1]. In the present multi-centre operational laboratory study it is shown that the FDA approved three-band immunochromographic RDT Binax NOW^® ^Malaria Test allows a semi-quantitative assessment of parasitaemia and rapid exclusion of high *P. falciparum *parasitaemia, which may facilitate clinical decision making in the acute care setting.

## Methods

In order to assess the utility of this RDT as a semi-quantitative measure of *P. falciparum *load in routine clinical practice, an operational laboratory study was conducted at two hospital-based laboratories with expertise in malaria diagnosis in The Netherlands (Academic Medical Center, Amsterdam, The Netherlands; Harbour Hospital, Rotterdam, The Netherlands). Of all patients detailed demographic, clinical and laboratory data were available, as well as the outcome measures severe malaria and death. Severe malaria was diagnosed according to predefined WHO criteria in travellers [7]. In both Dutch centres, parasitaemia was examined using the same protocol. Thick and thin smears were stained with Giemsa (Giemsa improved R66 Gurr, BDH, diluted 1:10, PH 7,2, 30 min). For an initial estimate of the parasite load, malaria thin smears were examined by light microscopy (100× objective and 12,5 ocular lens). If the parasitaemia was assumed to be ≤0.5% infected red blood cells, the exact parasite load was determined by counting the number of asexual parasites per 100 leukocytes in a thick smear. In case the initial parasitaemia was assumed to be 0.5-2.0%, the number of infected red blood cells was counted in 10 visual fields of a thin smear. The number of red cells per microscopic field in a thin smear was pre-calculated for the different microscopes in use. In case the initial parasitaemia was assumed to be >2.0%, the number of infected red blood cells was determined using a special ocular lens with a visual field area reduced to approximately 25%. Within this limited field of view both the total number of red blood cells and the number of infected red blood cells were counted in at least 10 visual fields. All counts were performed in duplicate and the final count was given as the average. In case of a discrepancy of >15% between the duplicate counts, a third count was performed. The number of asexual parasites/μl was finally calculated using the actual number of erythrocytes or leukocytes in a blood sample.

**Figure 1:**
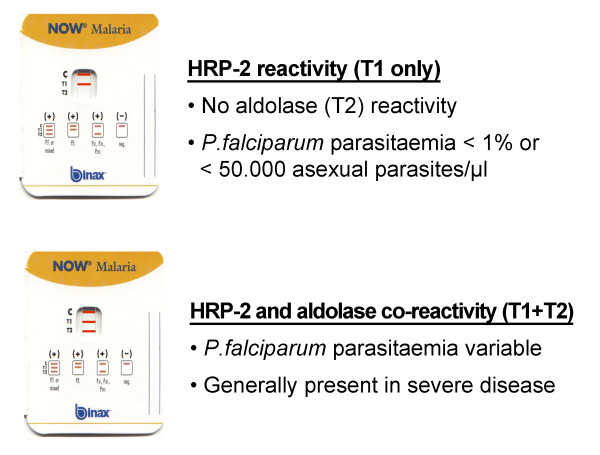
**Interpretation of the Binax NOW^® ^Malaria test results in patients with *P. falciparum *infection, related to microscopic parasitaemia**.

The RDTs were performed on fresh blood samples, simultaneously with microscopy of the blood slides. Every RDT and blood slide was read by two independent, experienced laboratory technicians. The Binax NOW^® ^Malaria Test was used as RDT and performed according to the manufacturer's instructions. The Binax NOW^® ^Malaria Test uses monoclonal antibodies that target the histidine-rich protein 2 (HRP-2) antigen specific to *P. falciparum *(the 'T1' line) and the pan-malarial antigen aldolase (the 'T2' line), common to all five *Plasmodium *species that can be detected in humans (*P. falciparum*, *Plasmodium vivax*, *Plasmodium ovale*, *Plasmodium malariae *and *Plasmodium knowlesi*) [8]. Aldolase co-reactivity was defined as both a reactive HRP-2 line (T1 line) as well as a reactive aldolase line (T2 line). Absence of aldolase co-reactivity was defined as a non-reactive aldolase (T2) but a reactive HRP-2 (T1) line (Figure 1).

For external validation of the reproducibility of the RDT findings in the Dutch centers, a reference center for malaria diagnosis participated (Institute of Tropical Medicine (ITM), Antwerp, Belgium). In this setting in thick and thin blood films, stained with Giemsa (Merck 1.09204.0500, diluted to 3.5% in pH 8.0, 20 min.), parasite densities were determined by counting asexual parasites against 200 white blood cells (WBC) in thick blood films and converting this number to parasites/μl using the actual white blood cell count [9]. Parasite densities were next converted to % infected red blood cells using the red blood cell count. For the purpose of this study, ITM provided RDT findings in relation to the parasite loads of 73 consecutive patients, but did not contribute to the clinical data.

## Results

A total of 257 patients with *P. falciparum *malaria were included. All patients had HRP-2 reactivity in the Binax NOW^® ^Malaria Test. The general characteristics and laboratory findings of 184 evaluable patients from Amsterdam (Academic Medical Center) and Rotterdam (Harbour Hospital) are provided in Table 1. The 23 patients with severe malaria presented with impaired consciousness in seven (three of them had a Glasgow Coma Score score < 11), severe anaemia in two, hyperbilirubinaemia in 12 and renal failure in three cases. Eleven patients had a parasitaemia > 5% (4 of them had a parasitaemia > 10%). Five patients received haemodialysis and one patient died. Of the 73 Belgian patients no clinical data were available.

**Table 1:** General characteristics and laboratory findings at initial clinical assessment of 184 patients with *P. falciparum *malaria

Parameter	Amsterdam (AMC)	Rotterdam (HZH)
Number of patients, *n*	77	107
Male/female	52/25	78/29
Age, median (range,) *yrs*	37 (0 - 61)	40 (16 - 70)
**Purpose of travel**		
Immigrant, *n (%)*	4 (5.2)	0 (0.0)
Visiting friends and relatives, *n (%)*	48 (62.3)	36 (33.6)
Tourist, *n (%)*	6 (7.8)	20 (18.7)
Business, *n (%)*	10(13.0)	27 (25.2)
Expatriate, *n (%)*	0 (0.0)	4 (3.7)
Sailor, *n (%)*	0 (0.0)	4 (3.7)
Unknown, *n (%)*	9 (11.7)	11 (10.3)
**Continent of acquisition**		
Africa, *n (%) *[West-Africa, *n (*%)]	73 (94.8) [63 (81.8)]	101 (94.9) [71 (66.4)]
South-America, *n (%)*	2 (2.6)	1 (0.9)
South East Asia, *n (%)*	2 (2.6)	7 (6.5)
**Clinical assessment**		
Severe malaria, *n (%)*	7 (9.1)	16 (15.0)
Non-severe malaria, *n (%)*	70 (90.9)	91 (85.0)
**Laboratory findings**		
Parasite load, median range, *trophozoites/μL*	4648 (50 - 558000)	10664 (2 - 1380600)
Malaria Now	77 (100)	107 (100)
HRP-2 and aldolase co-reactivity, *n (%)*	54 (70.1)	78 (72.9)
HRP-2 reactivity, *n (%)*	23 (29.9)	29 (27.1)

**Figure 2:**
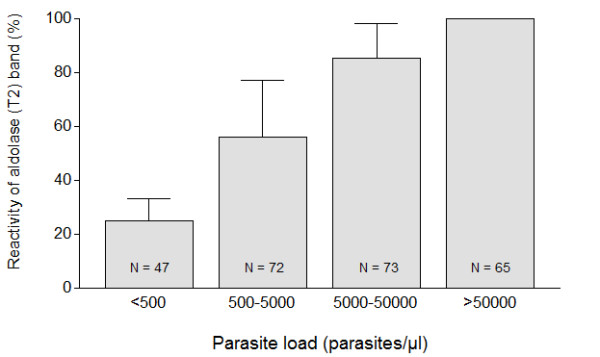
**Co-reactivity of aldolase and HRP-2 bands in Binax NOW^® ^Malaria rapid diagnostic test in relation to *P. falciparum *parasitaemia. Aldolase co-reactivity was consistently present at a parasitaemia above 50,000 asexual parasites/μl, but variably present at lower parasitaemia indicating that absence of aldolase co-reactivity always was associated with a parasitaemia ≤ asexual 50,000 parasites/μl. The RDT findings (absence or presence of aldolase co-reactivity) of the participating three centres are shown in relation to category of parasitaemia and expressed as mean ± SD**. The number of patients per category of parasitaemia is given within each respective bar.

Co-reactivity of HRP-2 and aldolase was observed in blood specimens with both low and high parasitaemia, ranging from 56 to 558,000 (Amsterdam), 23 to 1,380,600 (Rotterdam) and 26 to 400,000 asexual parasites/μl (Antwerp), respectively. The proportion of aldolase co-reactivity substantially increased with increasing parasitaemia (Figure 2). Aldolase co-reactivity was always present when parasitaemia was above 50,000 asexual parasites/μl (corresponding to approximately 1% parasitized erythrocytes). This observation was valid not only in the centers in Rotterdam and Amsterdam, but also in the laboratory setting of Antwerp. All patients with severe malaria (n = 23) invariably showed HRP-2 and aldolase co-reactivity. Hence, aldolase co-reactivity with HRP-2 had a sensitivity of 100% (95% confidence interval 85-100%) for severe malaria, but a poor positive predictive value (PPV) of only 21% (95% CI 11-25%), since aldolase co-reactivity was also present in 109 patients with uncomplicated malaria. In contrast, absence of aldolase reactivity (n = 52) had a negative predictive value (NPV) of 100% (95% CI 93 - 100%) for severe malaria. The implications of the RDT test outcomes for clinical decision-making are shown in Figure 1.

## Discussion

The results of the present study indicate that the Binax NOW^® ^Malaria Test allows a semi-quantitative assessment of parasitaemia in travellers returning with *P. falciparum *malaria.

HRP-2 reactivity (T1) in absence of aldolase reactivity, proved a reliable predictive marker for a low (< 1%) *P. falciparum *parasitaemia. In the current study population HRP-2 reactivity without aldolase reactivity was applicable to 30% of returning travellers with *P. falciparum*. When clinical findings and routine laboratory results in these patients also are not indicative of severe disease, the patient most likely has uncomplicated malaria. This knowledge is important for further clinical decision-making.

Co-reactivity of aldolase and HRP-2 was present in all patients with a parasitaemia above 50,000 asexual parasites/μl (corresponding to approximately 1% infected red blood cells). Others reported co-reactivity with Binax NOW^® ^in 80% of patients with *P. falciparum *parasitaemia > 40,000 parasites/μl [9]. The authors suggested co-reactivity of HRP-2 and aldolase possibly could function as a semi-quantitative marker of high *P. falciparum *parasitaemia [10]. In the present study this relation proved, however, not straightforward with co-reactivity of HRP-2 and aldolase also being present in patients with low parasitaemia (i.e. < 0.5%). As such co-reactivity of HRP-2 and aldolase is less reliable as marker for high parasitaemia.

The data of the present study suggest that aldolase and HRP-2 co-reactivity is present in all patients with severe malaria. Apparently HRP-2 and aldolase reactivity, which depends on the load of these antigens in the blood specimen, is preserved because these antigens are derived not only from circulating viable and non-viable malaria parasites, but also from sequestered parasites that are abundantly present in severe malaria. As a consequence of this sequestration, microscopic determination of peripheral blood smears might underestimate the total parasitaemia.

The relation between aldolase reactivity and *P. falciparum *parasitaemia, as observed with Binax NOW^® ^in this study, could be dependent of the process of manufacturing. Other RDTs with a similar three-band configuration, therefore, should be studied in detail, to assess about the precise relationship between *P. falciparum *parasitaemia and aldolase reactivity.

HRP-2 and aldolase co-reactivity may also be a feature of a mixed *Plasmodium *infection. In a recent study of 2,847 cases of imported malaria in the Netherlands [11], 75% of the infections were solely caused by *P. falciparum *whereas the remainder was caused by *P. vivax *(15%), *P. ovale *(7%), and *P. malariae *(3%), respectively. Only 0,7% of all infections was attributable to mixtures of species, mostly involving *P. falciparum*. Thus, in the Dutch setting, HRP-2 and aldolase co-reactivity is far more likely to reflect a mono-parasitic *P. falciparum *infection rather than a mixed infection.

Results of RDT's may facilitate clinical decision making in patients suspected of having malaria. There are however also some drawbacks to consider. First, these tests cannot replace clinical assessment of the ill-returning patient and results of RDT tests should always be confirmed by thin or thick blood smears, including parasite counts in case of *P. falciparum *malaria [6,12]. Second, the diagnostic power of this RDT test is dependent on the epidemiological setting, in particular the prevalence of the disease. The current findings may not simply be extrapolated to regions of malaria endemicity where low-grade malaria infections are far more prevalent and empirical anti-malarial treatment is common use, which may lead to false-negative and false-positive RDT findings, respectively. In addition, the majority of the travellers in this study contracted *P. falciparum *infection in Africa; other malaria-endemic continents like South-East Asia and South-America were underrepresented. Caution is warranted with extrapolating the applicability of the current findings to imported malaria acquired outside Africa. False negative results have been suggested for certain genetic polymorphisms of HRP-2 geographically confined to the Asia-Pacific region [13] and for *P. falciparum *isolates from South America lacking HRP-2 [14]. In addition, false negative test results may occur at high parasitaemia due to a so-called prozone effect, defined as false-negative or false-low results in immunological reactions due to an excess of either antigens or antibodies. The prozone effect was observed for HRP-2 in 16 of 17 RDTs (including the Binax NOW^® ^Malaria Test), resulting in a false low HRP-2 signal, whereas aldolase reactivity was not affected [15]. Finally, the clinician must also consider the possibility of a *P. knowlesi *infection, which may give rise to severe disease and fatal complications as well [8]. Even though early reports suggested that RDTs may not detect *P. knowlesi *infections, later studies demonstrated that *P. knowlesi *was reactive with the aldolase band in the Binax NOW^® ^Malaria Test, but not with HRP-2 and that aldolase reactivity depended on the *P. knowlesi *parasitaemia [8].

## Conclusion

In conclusion, the RDT Binax NOW^® ^Malaria Test allows a rapid semi-quantitative assessment of *P. falciparum *load in travellers with malaria returning from the tropics, especially for exclusion of high (>1%) parasitaemia in the acute care setting. This may facilitate clinical decision making for subsequent oral anti-malarial treatment or timely referral to a specialized centre for high-level monitoring and intensified parenteral treatment.

## Competing interests

The authors declare that they have no competing interests.

## Authors' contributions

TvG and PvG designed the study, acquired, analysed and interpreted data, drafted and revised the manuscript, MvW acquired and analysed data, and revised the manuscript, RK, PvT, JJ and JvH acquired, analysed and interpreted data, and revised the manuscript. All authors read and approved the final manuscript.
